# Pancreatic injury in children: a case report and review of the literature

**DOI:** 10.1186/s13256-017-1383-3

**Published:** 2017-09-09

**Authors:** Fayza Haider, Mohammed Amin Al Awadhi, Eizat Abrar, Mooza Al Dossari, Hasan Isa, Husain Nasser, Hakima Al Hashimi, Sharif Al Arayedh

**Affiliations:** 1 Pediatric Surgery Unit-Department of Surgery, Salmaniya Medical Complex, P.O. Box 12, Manama, Kingdom of Bahrain; 2 Department of Pediatrics, Salmaniya Medical Complex, Manama, Kingdom of Bahrain; 3 Department of Radiology, Salmaniya Medical Complex, Manama, Kingdom of Bahrain

**Keywords:** Case report, Blunt trauma abdomen, Pancreatic injury, Computed tomography, Liver injury, Pancreatic pseudocyst, Total parenteral nutrition, Non-operative management

## Abstract

**Background:**

Trauma is the main cause of morbidity and mortality in the pediatric population. Blunt trauma to the abdomen accounts for the majority of abdominal injuries in children. Pancreatic injury, although uncommon (2 to 9%), is the fourth most common solid organ injury. Unlike other solid organ injuries, pancreatic trauma may be subtle and difficult to diagnose. Computed tomography currently is the imaging modality of choice.

As the incidence of pancreatic injury in children sustaining blunt abdominal trauma is low, management remains a challenge.

**Case presentation:**

We present a 7-year-old Bahraini boy who sustained blunt trauma to his abdomen. He presented with abdominal pain and vomiting. His examination revealed abdominal distension and an epigastric bruise. Contrast-enhanced computed tomography reported grade III liver injury, grade I bilateral renal injury, a suspicion of splenic injury, and a grade III to IV pancreatic injury. He was admitted to Pediatric Intensive Care Unit and was treated conservatively. Because he was stable, he was discharged to the surgical ward at day 3. At day 18 he developed a pancreatic pseudocyst that was aspirated and recurred at day 25 when a pigtail catheter was inserted. He was kept on total parenteral nutrition through a peripherally inserted central catheter. The pigtail catheter was removed on day 36 and a low fat diet was started by day 44. He was discharged home at day 55 in good health. Out-patient follow-up and serial abdominal ultrasound showed resolution of the cyst and normalization of blood tests.

**Conclusion:**

Non-operative management of pancreatic injury is effective and safe in hemodynamically stable patients with no other indication for surgery.

## Background

Blunt trauma to the abdomen accounts for the majority of abdominal injuries in children. Pancreatic injury, although uncommon (2 to 9%) [1], is the fourth most common solid organ injury, following the spleen, liver, and kidneys [2]. The first description of a pancreatic injury was by Travers in 1827 in England [3].

Unlike other solid organ injuries, pancreatic trauma may be subtle or difficult to visualize on computed tomography (CT) immediately after the incident. The radiologist often must rely on secondary findings in the absence of an obvious laceration or frank fracture of the pancreas [3].

As the incidence is low, management remains a challenge. In the past two decades, there has been ongoing debate about the optimal approach to treating pancreatic injuries with some authors advocating early operative intervention and others suggesting that a non-operative approach is advantageous and safe [1]. Non-operative management of minor pancreatic injury is well accepted, but the management of more serious pancreatic injury with capsular, ductal, or parenchymal disruption remains controversial [2]. Here, we report a case of severe blunt pancreatic injury with ductal injury that was successfully managed non-operatively.

## Case presentation

**Fig. 1:**
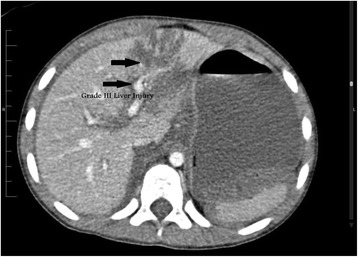
Axial post-intravenous contrast-enhanced computed tomography scan shows grade III liver laceration at segment IV of the liver in a 7-year-old boy with a blunt abdominal trauma (*arrows*)

**Fig. 2:**
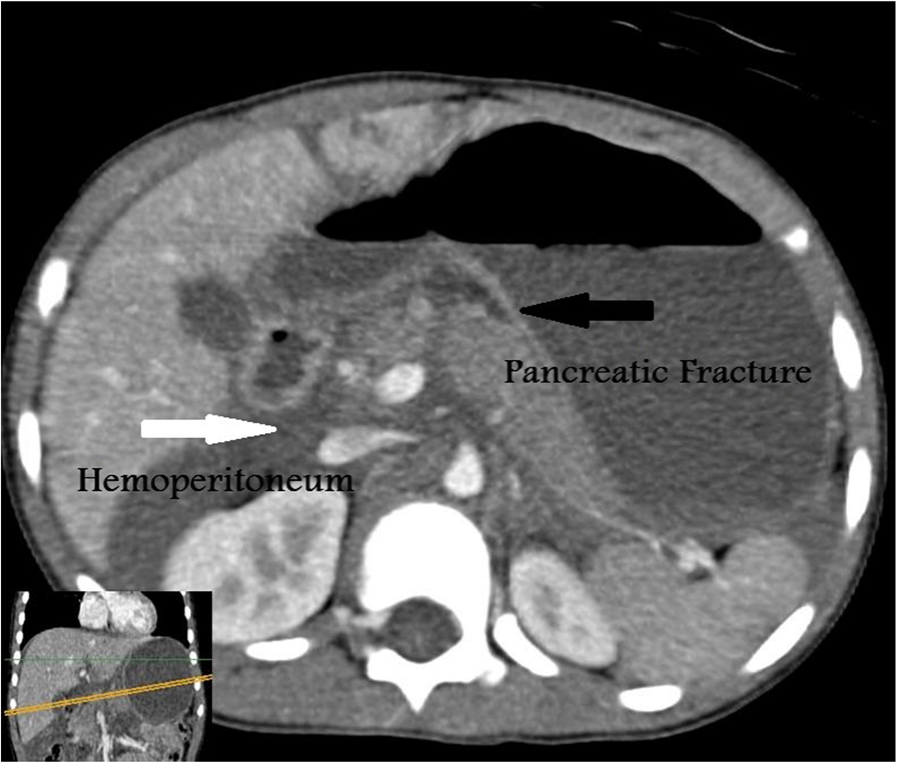
Intravenous contrast-enhanced computed tomography scan shows vertical hypodense transection line through the pancreatic neck (*black arrow*) and hemoperitoneum (*white arrow*) fills the retroperitoneal spaces in a 7-year-old boy with a blunt abdominal trauma

**Fig. 3:**
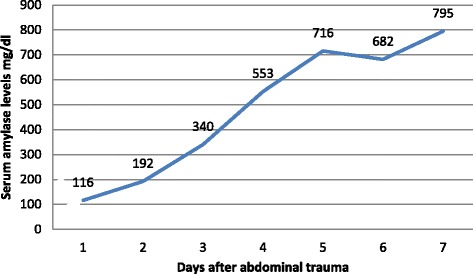
Gradual rise in serum amylase levels during the first week of injury in a 7-year-old boy with a blunt abdominal trauma

**Fig. 4:**
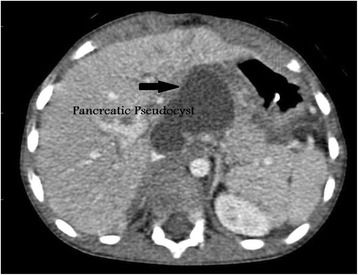
A post contrast axial computed tomography scan at portovenous phase shows a pseudocyst (*black arrow*) formation at the lesser sac in a 7-year-old boy with a blunt abdominal trauma. The liver laceration has healed

**Fig. 5:**
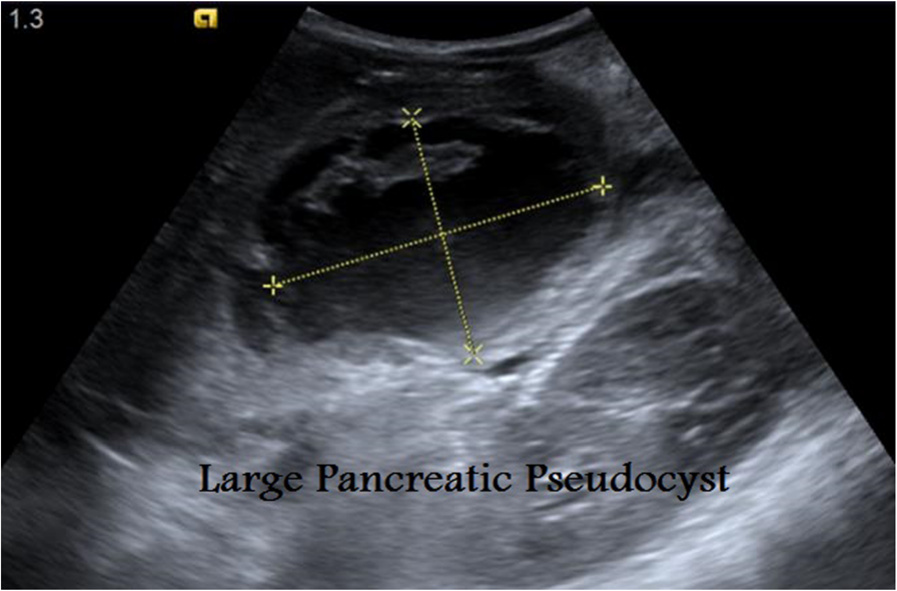
Ultrasound of the abdomen showing large pseudocyst containing echogenic material in a 7-year-old boy following a blunt abdominal trauma with pancreatic injury

**Fig. 6:**
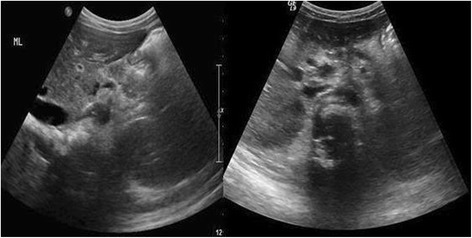
Axial ultrasound images at the level of the pancreas and retroperitoneum shows complete healing of the pancreatic injury and resolution of the lesser sac pseudocyst in a 7-year-old boy with a blunt abdominal trauma

**Fig. 7:**
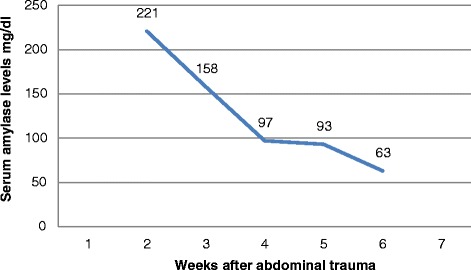
Normalization of serum amylase in a 7-year-old boy with a blunt abdominal trauma and pancreatic injury

This patient is a 7-year-old Bahraini boy who sustained blunt trauma to his abdomen from the bar of a football goal post. He presented with abdominal pain and vomiting. On arrival to accident and emergency department, he was conscious, alert, and oriented. His pulse was 121 beats per minute, blood pressure was 96/53 (mean 68) mmHg, and respiratory rate was 30 cycles per minute. His abdomen was distended with an epigastric bruise. On admission his hemoglobin was 9.2 g/dL (12 to 14.5), white blood cell count was 20.3×10^9^/L (3.6 to 9.6), serum amylase was 116 U/L (30 to 118), and glucose was 26.8 mmol/L (3.6 to 8.9; Table 1). Pan-CT revealed a grade III liver injury (Fig. 1), grade I bilateral renal injury, and a suspicion of splenic injury was raised by the radiology registrar. The CT films were reviewed next day by a consultant radiologist and reported a grade III to IV pancreatic injury (Fig. 2). Our patient was admitted to the Pediatric Intensive Care Unit (PICU) and was treated conservatively. Because he was stable, he was discharged to the surgical ward by day 3. His blood test results were monitored closely and showed a gradual rise in serum amylase level during the first week of injury (Fig. 3). At day 18 he developed abdominal distension and vomiting. An abdominal examination revealed a palpable epigastric mass. A CT scan showed a pseudocyst (Fig. 4) which was aspirated percutaneously under ultrasound guidance by an interventional radiologist. The cyst recurred at day 25 (Fig. 5) so a pigtail catheter was inserted for continuous drainage under ultrasound guidance. Throughout his stay, he was maintained on total parenteral nutrition (TPN) through a peripherally inserted central catheter (PICC) line. The pigtail catheter was removed after ten days and a low fat diet was started by day 44 as he did not tolerate orally administered nutrition earlier. He was discharged home at day 55 in good health. Out-patient follow-up and serial ultrasound showed resolution of the cyst (Fig. 6) and normalization of his blood investigations (Fig. 7 and Table 1).

**Table 1:** Monitored blood investigations during the first week until follow-up visit after blunt abdominal trauma with pancreatic injury in a 7-year-old boy

Test	D1	D2	D3	D4	D5	D6	D7	W2	W3	W4	W5	FU
Hemoglobin (12–14.5 g/dL)	9.2	13.9	11.5	11.2	9.9	10.4	11.9	10.3	9.4	9.0	8.9	11.1
White blood cells count (3.6–9.6 ×10^9^/L)	20.3	6.4	5.2	9.2	11.9	12.2	14.3	3.8	4.3	5.6	5.2	5.4
Platelets (150–400×10^9^/L)	148	154	121	95	151	169	218	93	82	75	190	299
Glucose (3.6–8.9 mmol/L)	26.8	6.8	5.9	5.2	5.5	4.6	5.8	7.0	6.5	6.6	4.8	5.8
Alkaline phosphatase (50–136 U/L)	239	190	135	122	130	106	135	146	129	154	154	218
Alanine amino transferase (<41 U/L)	1327	955	1243	1247	807	599	447	30	30	77	52	39
G-glutamyl transferase (15–85 U/L)	33	36	29	28	31	30	40	32	27	43	42	31
Amylase (30–118 U/L)	116	192	340	553	716	682	795	221	158	97	93	63

*D* day, *W* week, *FU* follow-up

## Discussion

Blunt abdominal trauma is the major cause of abdominal injury in children. Whereas injury to the pancreas is uncommon; it is the fourth most common solid organ injury. Death directly attributable to pancreatic injury in children has not been reported previously in the English language literature. Traditionally, pancreatic injury has been difficult to diagnose and treat, with diagnostic and therapeutic surgical interventions necessarily common [4].

The mechanism of injury is attributed to the compression of the pancreas against the rigid spinal column or by discrete intrusion forces. Young children with flatter diaphragms, thinner abdominal walls, and higher costal margins sustain pancreatic injuries from blows to the abdomen more than adults [2]. This feature is evident in our presented patient as he was thin built.

As all patients who have experienced trauma undergo CT to detect injuries, the sensitivity of abdominal CT in pancreatic injury is unknown and false-positive and false-negative evaluations are common. Problems relating to streak artifacts, un-opacified loops of bowel, and observer error persist. Trauma to adjacent organs, such as the spleen and kidneys, can further obscure evaluation of the pancreas [3].

Lane *et al.* reported that the actual pancreatic laceration is difficult to identify [3]. However, they mentioned that a careful evaluation of the reported secondary findings (that is, intrapancreatic hematoma, intraperitoneal and extraperitoneal fluid, fluid separating the splenic vein and pancreatic body, and a thickened left anterior renal fascia), often should lead the radiologist to the site of fracture once there is a suspicion and this definitely requires experience [3]. The initial CT report of our patient did not detect the pancreatic injury until an experienced consultant reviewed the films and detected the pancreatic injury.

The diagnostic relevance of CT is limited. CT in combination with magnetic resonance cholangiopancreatography (MRCP) may be a better option for exclusion of pancreatic duct lesions [5]. Not all centers have the expertise to do endoscopic retrograde cholangiopancreatography (ERCP) for pediatric patients; hence, they must rely on the CT findings and the experience of the radiologist which we did as we do not do ERCP for younger children.

Although both the sensitivity and specificity of an elevated admission amylase have been shown to be low, the relationship between repeat serum amylase and injury severity was shown to have strong statistical significance [2]. Serum amylase level may not be diagnostic within three or fewer hours after trauma [6]. Aydogdu *et al*. studied their patients with pancreatic injury and compared the ones who developed pseudocyst to the ones who did not develop it and found that a rise in the serum amylase level may be a good predictor for the development of pancreatic pseudocyst [7]. This was shown clearly in our patient where his serum amylase level was normal on admission and increased gradually over the first week of admission and thereafter he developed a pancreatic pseudocyst (Fig. 3).

Even at a single institution, the decision to operate for pancreatic trauma appears to be case dependent. This variability highlights the need for more rigorous outcomes research to guide management decisions [1].

Authors advocating for operative treatment of pancreatic trauma argue that non-operative treatment results in unnecessary prolongation of hospitalization, inconvenience to patients, and increased incidence of pancreatic pseudocyst, particularly when ductal injury is suspected. Other authors have argued for a conservative approach to major pancreatic trauma with selective operative management. Some recommended distal pancreatectomy in children with grade II injury but non-operative management of grade III, suggesting that pseudocyst formation should be considered a “favorable” outcome in the natural history of traumatic pancreatic injury [1]. Our patient developed a pseudocyst that was drained percutaneously initially and then required insertion of a pigtail catheter for continuous drainage by an interventional radiologist. This relieved the child’s symptoms and helped him in his recovery.

High-grade pancreatic injuries in children are rare and significant variability exists in non-operative management strategies, which may affect outcomes and effective resource utilization where length of stay is always a concern [8, 9]. Recently published studies show that children managed non-operatively have equivalent and sometimes better outcomes when compared with operative management in regard to death and overall complications [8–10].

## Conclusions

Pancreatic injury although uncommon can occur and warrants special attention due to its associated morbidities.

Although case dependent, non-operative management is the treatment of choice in solid organ injuries and pancreas is added to the list. This management can be applied to all grades of pancreatic injury provided the child remains hemodynamically stable.
